# Super high-flux continuous hemodialysis: an efficient compromise for blood purification in sepsis

**DOI:** 10.1186/cc10984

**Published:** 2012-03-20

**Authors:** T Rimmelé, M Page, C Ber, F Christin, J Baillon, J Crozon, C Chapuis-Cellier, R Ecochard, B Allaouchiche

**Affiliations:** 1 Edouard Herriot Hospital, Hospices Civils de Lyon, France

## Introduction

High cut-off membranes are proposed for blood purification therapy in septic shock. However, albumin loss related to these membranes is a major drawback limiting their clinical acceptance. Super High-Flux membranes with an optimized cut-off may combine enhanced middle molecule clearances (inflammatory mediators) with limited albumin loss. The aim of our study was to compare small, middle molecule clearances and albumin loss between continuous hemodialysis using a Super High-Flux membrane (SHF-HD) and conventional continuous hemofiltration (CVVH).

## Methods

After approval by the ethics committee, patients were enrolled in a single-blind RCT. Patients with septic shock and acute kidney injury received either SHF-HD (EMiC2^® ^filter; Fresenius Medical Care) (cut-off = 40 kDa, dialysate flow rate of 40 ml/kg/hour) or conventional CVVH (cut-off = 30 kDa, UF flow rate of 40 ml/kg/hour). Each patient received a maximum of three sessions of 48 hours each. Creatinine (113 Da), β_2_-microglobulin (β_2_-M) (11.8 kDa), kappa free light chain of immunoglobulins (κ-FLC) (23 kDa) and albumin (68 kDa) clearances were measured at 15 minutes, 1 hour, 4 hours, 12 hours, 24 hours and 48 hours. β_2_-M and κ-FLC were chosen as a middle molecular weight marker. A linear mixed-effects model compared clearances between groups.

## Results

**Figure 1:**
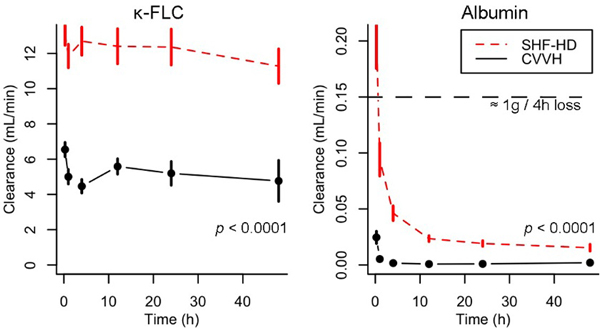


Twenty-four patients were included, 12 in the SHF-HD group (32 sessions) and 12 in CVVH (30 sessions). κ-FLC and albumin clearances were higher in the SHF-HD group over time. No difference was observed for creatinine (*P *= 0.18) and β_2_-M (*P *= 0.98) clearances. Plasma albumin levels and the amount of albumin infused did not differ between groups. See Figure 1.

## Conclusion

The removal of middle molecular weight molecules is higher with SHF-HD. Albumin loss was limited in both groups, even with SHF-HD. Therefore, SHF membranes seem to represent an alternative to high cut-off membranes for blood purification therapies.

